# Effects of Blood Transportation on Human Peripheral Mononuclear Cell Yield, Phenotype and Function: Implications for Immune Cell Biobanking

**DOI:** 10.1371/journal.pone.0115920

**Published:** 2014-12-26

**Authors:** Anita Posevitz-Fejfár, Vilmos Posevitz, Catharina C. Gross, Urvashi Bhatia, Frank Kurth, Verena Schütte, Amit Bar-Or, Sven G. Meuth, Heinz Wiendl

**Affiliations:** 1 University Hospital Muenster, Department of Neurology, Albert-Schweitzer-Campus 1, Muenster, Germany; 2 Montreal Neurological Institute and Hospital, Department of Neurology and Neurosurgery, McGill University, Montreal, Quebec, Canada; New York University, United States of America

## Abstract

Human biospecimen collection, processing and preservation are rapidly emerging subjects providing essential support to clinical as well as basic researchers. Unlike collection of other biospecimens (e.g. DNA and serum), biobanking of viable immune cells, such as peripheral blood mononuclear cells (PBMC) and/or isolated immune cell subsets is still in its infancy. While certain aspects of processing and freezing conditions have been studied in the past years, little is known about the effect of blood transportation on immune cell survival, phenotype and specific functions. However, especially for multicentric and cooperative projects it is vital to precisely know those effects. In this study we investigated the effect of blood shipping and pre-processing delay on immune cell phenotype and function both on cellular and subcellular levels. Peripheral blood was collected from healthy volunteers (n = 9): at a distal location (shipped overnight) and in the central laboratory (processed immediately). PBMC were processed in the central laboratory and analyzed post-cryopreservation. We analyzed yield, major immune subset distribution, proliferative capacity of T cells, cytokine pattern and T-cell receptor signal transduction. Results show that overnight transportation of blood samples does not globally compromise T- cell subsets as they largely retain their phenotype and proliferative capacity. However, NK and B cell frequencies, the production of certain PBMC-derived cytokines and IL-6 mediated cytokine signaling pathway are altered due to transportation. Various control experiments have been carried out to compare issues related to shipping versus pre-processing delay on site. Our results suggest the implementation of appropriate controls when using multicenter logistics for blood transportation aiming at subsequent isolation of viable immune cells, e.g. in multicenter clinical trials or studies analyzing immune cells/subsets. One important conclusion might be that despite changes due to overnight shipment, highly standardized central processing (and analysis) could be superior to multicentric de-central processing with more difficult standardization.

## Introduction

Characterization and analysis of human blood and immune cell phenotype and function is becoming more and more important both for experimental and clinical studies: among others, this is relevant to investigating the mechanisms of action of immune therapies, monitoring immune function or addressing basic scientific questions related to the etiopathogenesis of various diseases and/or their therapeutic targeting. Analysis of peripheral immune response is essential for assessing response patterns and better understanding treatment and/or disease-induced immunological effects and immune competence, as well as for validating the clinical relevance of newly discovered biomarkers. All these aspects require high quality blood samples allowing isolation of viable and functionally unaltered immune cells for further experimental analysis to make precise observations and thereby reliable conclusions. Clinical trials are typically conducted in a multicenter setting. Therefore investigators often face the logistical challenge of shipping blood samples to remote locations (e.g. central laboratories). Experimental studies assessing peripheral immune cell function meanwhile are also often multicentric, since e.g. paucity of samples requires multicentric collection. Central laboratories may provide a number of advantages such as qualified personnel, SOP-based standardized sample processing, and minimal experimental variation. Likewise, centralized biobank facilities specialized for biospecimen collection, ensure controlled transport, cryopreservation, and regular quality assessment of collected biospecimen and thereby guarantee good quality biomaterial benefiting both clinical and basic researchers.

Currently, multicenter clinical trials rely typically on commercial courier services to transport blood to central (academic and non-academic) laboratories. Very generally speaking, academic multicenter collaborations on cellular biobanking are usually less professionally organized, mostly because of cost issues influencing feasibility.

Despite of the ever expanding demand for human PBMC, the effects of environmental factors (such as temperature changes, duration of transport) on the physiology of immune cells has not yet been thoroughly investigated. Understanding how varying shipping conditions induce alterations, which influence immune phenotype and function, can help to assess confounding variations affecting data quality and interpretation.

At present several studies have investigated the effect of physical factors during or post-transportation such as environmental conditions, packaging material, delayed processing of shipped blood samples and cryopreservation [1]–[14]. Importantly some studies do report an altered immune cell function due to mishandling or suboptimal conditions during processing and/or storage [1], [2], [7], [8], [10], [15], [16]. These studies urge further steps to better understand (and thereby better control) the effect of transportation on immune cell populations and their function, enabling improvement of sample quality and thereby generation of reliable results.

While the above listed studies addressed the effect of some pre-analytical factors on shipped blood, they are limited to single read-outs (e.g. proliferation assay [1]) and/or focus on different physical parameters than the shipping itself: e.g. processing delay [2], [7], [8], centrifuging [8], cryopreservation and its duration [5], [10] [11], [12], [13], media used for cryo preservation [3], [4] [6], [14]). Our study bridges this gap by performing a simultaneous analysis of multiple transportation-induced effects on the quality of immune cells including phenotype and function (proliferation, cytokine production and signaling pathways). We believe that our data are also complementary to the observations made by Olson et al. [9] who studied the effect of temperature (during transportation or transportation simulating conditions) on viability and function of PBMC (reflected by one representative cytokine - IFNγ - in ELISPOT assay).

In order to precisely detect and differentiate transportation-induced effects on immune cells we drastically reduced the number of variables as follows: 1) we included healthy individuals who donated blood twice (locally and at a remote location) which allowed us to monitor transport-induced changes within the same individual and in parallel exclude any disease related effect(s); 2) we used equal shipping conditions (sampling tube, packaging, service provider); 3) all analyses were performed using standardized procedures in a central laboratory. For functional assessment of lymphocytes we have chosen read-outs representing a) standardizable assays (cytokine production), which are frequently used in clinical studies, b) functional readouts, which typically require specialized laboratories (proliferation assay), c) non-standardizable and yet research-laboratory linked methods (Western blot for analyzing signal transduction pathways). Taken together our study provides a comprehensive analysis of cellular and subcellular lymphocyte functions as well as immune cell subset-optimized guidelines helping to properly design clinical and/or immune monitoring studies that require transportation of blood samples to remote locations.

## Materials and Methods

### Sample collection and shipment

Blood was drawn into EDTA-containing tubes (K2E Vacutainer, BD) from healthy volunteers (n = 9, 3 female and 6 male, age: 28–45, mean age: 35) at the study center (“non-shipped”) and at one of two distal locations (“shipped”) from the same individuals. Material was transported with an express courier service overnight in foam insert-containing Vacuette transport containers (VTC, BD) at ambient temperature and processed in the central laboratory within 24 hours (time interval until processing: 17.7 h–23.3 h, with a mean of 21.8 h). Non-shipped samples were processed within 2 hours (5 min–2 h, with a mean of 41 min). The time interval between the two blood drawings of each individual was 4–10 days (average: 6.7 days) and none of the individuals showed or reported any disease symptoms at any of the sites.

### PBMC preparation and cryopreservation

All steps of PBMC preparation have been carried out at room temperature. The content of the blood collecting tubes from the same donor were pooled and mixed 1∶1 with pre-warmed (room temperature) 1×PBS (w/o Ca^2+^, Mg^2+^). The blood: PBS mixture was layered onto Lymphoprep (Axis-Shield) and subsequently centrifuged at 800 g for 30 min, at room temperature (slow acceleration, no brake). The ring-shape interphase (peripheral blood mononuclear cells, PBMC) was collected with a Pasteur pipette into a new 50 ml tube and diluted up to 50 ml with pre-warmed 1×PBS (w/o Ca^2+^, Mg^2+^) and centrifuged with 300 g for 10 min, at room temperature, with fast acceleration and with brake. The total cell number and the number of living cells were determined with Trypan blue exclusion. Following a further washing step in 1×PBS (300 g, 10 min, room temperature), cells were resuspended in CTLCABC-cryo freezing media (Immunospot). The components of the ABC media (CTL-Cryo A, CTL-Cryo B and CTL-Cryo C) were pre-warmed to room temperature prior mixing and adding the mixture to the cells. 1 ml aliquots with 1×10^7^ cells/ml were gradually frozen in Mr. Frosty device at −80°C for at least 48 hours (maximum 7 days) and transferred into a cryo tank (liquid nitrogen vapor phase).

Cellular yield was determined as number of living cells/ml of blood. Viability was defined as % of living cells/total cell count. Cell counts are based on Trypan blue exclusion.

### PBMC thawing

PBMC-containing cryo vials were placed into pre-warmed water bath (37°C) for 8 minutes. The cell suspension was mixed by gentle flipping, and the content of the vial was transferred into a 50 ml conical tube. 9 ml pre-warmed (37°C) FCS-supplemented RPMI media was added slowly (the first 4 ml drop-vice) while gently whirling the tube. The cell suspension was centrifuged with 1500 rpm (453 g), 10 min, at room temperature and the pellet was resuspended in pre-warmed cell culture media (volume and composition adjusted to the assay for which the cells were to be used). Cells were counted with Trypan blue exclusion and applied in subsequent assays. Based on the cell count recovery was calculated and provided as % of living cells, where the frozen amount (10^7^/vial) was considered as 100%.

### Multicolor flow cytometry

2×10^5^ freshly thawed PBMC were washed once in flow cytometry buffer (PBS supplemented with 2% heat inactivated FCS and 2 mM EDTA) for 4 min, with 290 g, at room temperature. PBMC were re-suspended in 100 µl of the appropriate antibody working solution (see Table 1) and diluted in flow cytometry buffer. Following 30 min staining at room temperature PBMC were washed once with flow cytometry buffer (4 min, 290 g, room temperature) and resuspended in 300 µl flow cytometry buffer. Cells were acquired (stop gate set to 100.000 leukocytes) with Navios flow cytometer (Beckman Coulter). A thorough compensation using also isotype-matched controls was performed before measuring the samples. Data were analyzed using KALUZA (Beckman Coulter) software.

**Table 1:** Antibody composition of staining master mix (basic panel and T_reg_ panel) applied for multicolor flow cytometry.

Antibody	Fluorochrome	Clone	Company	Dilution
Basic panel				
Anti-CD14	FITC	RM052	Beckman Coulter	1∶200
Anti-CD3	PC5.5	UCHT1	Beckman Coulter	1∶200
Anti-CD56	PC7	N901	Beckman Coulter	1∶200
Anti-CD4	APC	13B8.2	Beckman Coulter	1∶200
Anti-CD19	APC-A700	J3-119	Beckman Coulter	1∶200
Anti-CD8	Pacific Blue	B9.11	Beckman Coulter	1∶200
T_reg_ panel				
Anti-CD45RA	FITC	ALB11	Beckman Coulter	1∶200
Anti-CD27	PE		Beckman Coulter	1∶100
Anti-CD3	ECD	UCHT1	Beckman Coulter	1∶100
Anti-CD25	PC7	B1.49.9	Beckman Coulter	1∶200
Anti-CD56	APC	NCAM16.2	BD Biosciences	1∶100
Anti-CD127	APC-A700		Beckman Coulter	1∶200
Anti-CD4	Krome Orange	13B8.2	Beckman Coulter	1∶200

### PBMC proliferation assay

96-well cell culture plates were coated with 100 µl PBS/well or with 10 µg/ml anti-hCD3 (OKT3) antibody and incubated overnight at 4C°. Next day, freshly thawed PBMC (see *PBMC thawing*) were resuspended in 10 ml cell culture media (RPMI supplemented with 10% FCS) and counted with automatic cell counter. After a washing step in pre-warmed 1×PBS at room temperature (1500 rpm, 5 min), cells were loaded with eFluor670 cell proliferation dye (eBioscience) according to the manufacturer's instructions. Briefly: cells were labeled with 10 µM dye solution for 10 min at 37°C (protected from light). The reaction was stopped by adding 4–5 volumes of cold cell culture media and incubation for 5 min on ice. After a washing step cells were resuspended and 2×10^5^ dye-loaded PBMC were cultured in 200 µl cell culture media in the presence or absence of stimulating antibodies (1 µg/ml anti-hCD28 (soluble) +10 µg/ml plate bound anti-CD3 (OKT3)) in humidified incubator at 37°C for 6 days. A dye-loading control was set using 10^5^ cells, incubated for 1 hour at 37°C with eFluor670 and acquired immediately by flow cytometer. After 6 days cell culture plates were centrifuged (1500 rpm (453 g) for 5 min at 4°C) and the supernatant collected for cytokine measurement. Subsequently PBMC were resuspended in FACS buffer (PBS supplemented with 0.1% BSA and 0.1% NaN_3_) for washing and stained in 50 µl antibody master mix (1∶25 dilution of each): anti-hCD4-FITC (BioLegend), anti-hCD8-Pacific Blue (Beckman Coulter), anti-hCD3-PerCP/Cy5.5 (BioLegend), anti-hCD56-PC7 (Beckman Coulter), anti-hCD19-PE (BD Pharmingen) for 30 min at 4°C. After a washing step cells were resuspended in FACS buffer and acquired by flow cytometry (Gallios 3 L 10 C, Beckman Coulter) and analyzed using KALUZA software (Beckman Coulter).

### Cytokine measurement in PBMC supernatant

Cytokine levels were determined in supernatants collected from the PBMC proliferation assays (anti-CD3 + anti-CD28 stimulated cells) after 6 days. All cytokine measurements were carried out with the human Th1/Th2/Th9/Th17/Th22 13plex bead array Kit (eBioscience, BMS817FF) according to the manufacturer's instructions. Briefly, samples were incubated with a bead mixture and biotin-conjugated cytokine specific antibody mixture light protected for 2 hours at room temperature. Samples were washed twice by adding assay buffer and spinning down with 200 g for 5 min. Staining with Streptavidin-PE solution was carried out under light protected conditions for 1 hour at room temperature, followed by 2 washing steps. Cytokines were assessed by flow cytometry (Gallios 3 L 10C, Beckman Coulter) and analyzed with FlowCytomixPro software (eBioscience).

### Analysis of TCR-and cytokine receptor signaling

PBMC were stimulated either 5 min with 10 µg/ml anti-CD3 (OKT3) +1 µg/ml soluble anti-CD28 antibodies or 30 min with rhIL-6 (20 ng/ml) in RPMI at 37C°. Western blot of cell lysates was done as described previously [17], [18]. In brief: following stimulation, cells were washed in ice cold PBS and lysed immediately in lysis buffer containing 1% Nonidet P-40, 1% lauryl maltoside (N-dodecyl-D-maltoside), 50 mM Tris (pH 7.5), 165 mM NaCl, 10 mM EDTA, 10 mM NaF, 1 mM phenyl-methyl-sulfonyl-fluorid, and 1 mM Na3VO4. Post-nuclear lysates were subjected to SDS-PAGE and proteins were transferred onto nitrocellulose membranes (Whatman, 0.45 µm pore size). Membranes were treated with a blocking step in 5% milk for 1 hour to prevent unspecific antibody binding and washed in 1×TBS supplemented with 0.1% Tween-20, then probed with anti-pErk1/2 (Thr202/Tyr204 of Erk1 and Thr185/Tyr187 of Erk2, Cell Signaling; 42–44 kDa) to monitor T-cell receptor induced signaling and with anti-pSTAT-3 (Tyr 705, Cell Signaling; 80 kDa) for IL-6 induced signaling, both rabbit IgG antibodies. Equal protein loading was controlled by probing the membranes with β-actin antibody (mouse IgG, clone AC-15, Sigma, 42 kDa). For secondary staining the appropriate horseradish peroxidase-conjugated anti-rabbit and anti-mouse IgG antibodies were used (Jackson Immuno Research). For molecular weight sizing Precision Plus Protein Dual Colour Standard has been applied (Bio-Rad).

### Statistical analysis

All data are expressed as mean ± SEM. Statistical analysis was performed using paired Student's t test; p values <0.05 were considered significant; p values <0.01 were considered highly significant. For choosing the optimal statistical analysis method the Institute of Biometry and Clinical Research of the University of Muenster has been consulted.

### Study participants

All study participants were healthy volunteers and provided written informed consent. Collection of blood samples from healthy individuals was approved by the ethics committee of Westphalia-Lippe and the Medical Faculty at the University of Muenster (2010-262-f-S).

## Results

### Effect of sampling tubes on PBMC viability

**Figure 1:**
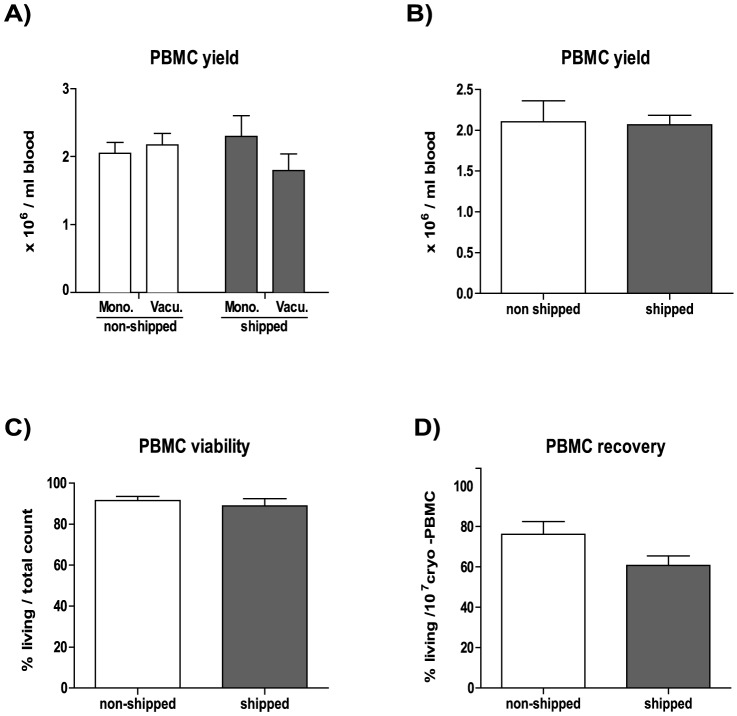
The effects of overnight shipping on PBMC yield and survival. The cell number of blood derived PBMC was determined based on Trypan blue exclusion. A) Blood was collected in S-Monovette (Sarsted) or K2E Vacutainer (BD) tubes from 4 donors on site or at another location. Shipped samples were transported in the collection tube. PBMC yield was calculated per volume (10^6^ living PBMC/ml blood). B) Yield (10^6^ living PBMC/ml blood) is shown for PBMC derived from non-shipped vs. shipped blood (n = 9) prior to freezing. C) Viability (% of living cells/total cell count) is shown for non-shipped vs. shipped samples (n = 9), prior to freezing. D) Post-cryopreservation recovery is calculated for thawed samples (non-shipped vs. shipped, n = 9) and is provided as percentage of living cells from 10^7^ total (frozen amount/vial).

Before investigating the effect of shipping on blood samples we ran a comparative test of different blood sampling tubes on the overall PBMC yield. We compared K_2_-EDTA buffer containing BD Vacutainer versus Sarstedt Monovette sampling tubes and found no significant differences in the overall PBMC yield (Fig. 1A). Based on this we decided to use the BD-Vacutainer system for our study, due to its frequent application in clinical studies.

### Effect of shipping on PBMC viability

First we investigated the effects of blood transportation on PBMC yield and viability. Blood was taken from n = 9 healthy donors and PBMC were isolated either within 2 hours (non-shipped) or after overnight transportation within 24 hours (shipped). Subsequently, the relative PBMC yield (number of PBMC/ml blood) and viability (number of living cells from total cell count) was assessed before cryopreservation. We observed that transportation ‘per se’ did not have any considerable influence on the PBMC yield and viability (Fig. 1B and 1C, respectively). Next, we investigated the effect of shipping on PBMC recovery after cryopreservation. To do so, we determined the number of living lymphocytes after thawing compared to total number of frozen cells per vial and found that cryopreservation and/or the thawing procedure resulted in reduced numbers of living cells (statistically not significant) in transported blood-derived samples (Fig. 1D).

### Effect of shipping on immune cell subset distribution

**Figure 2:**
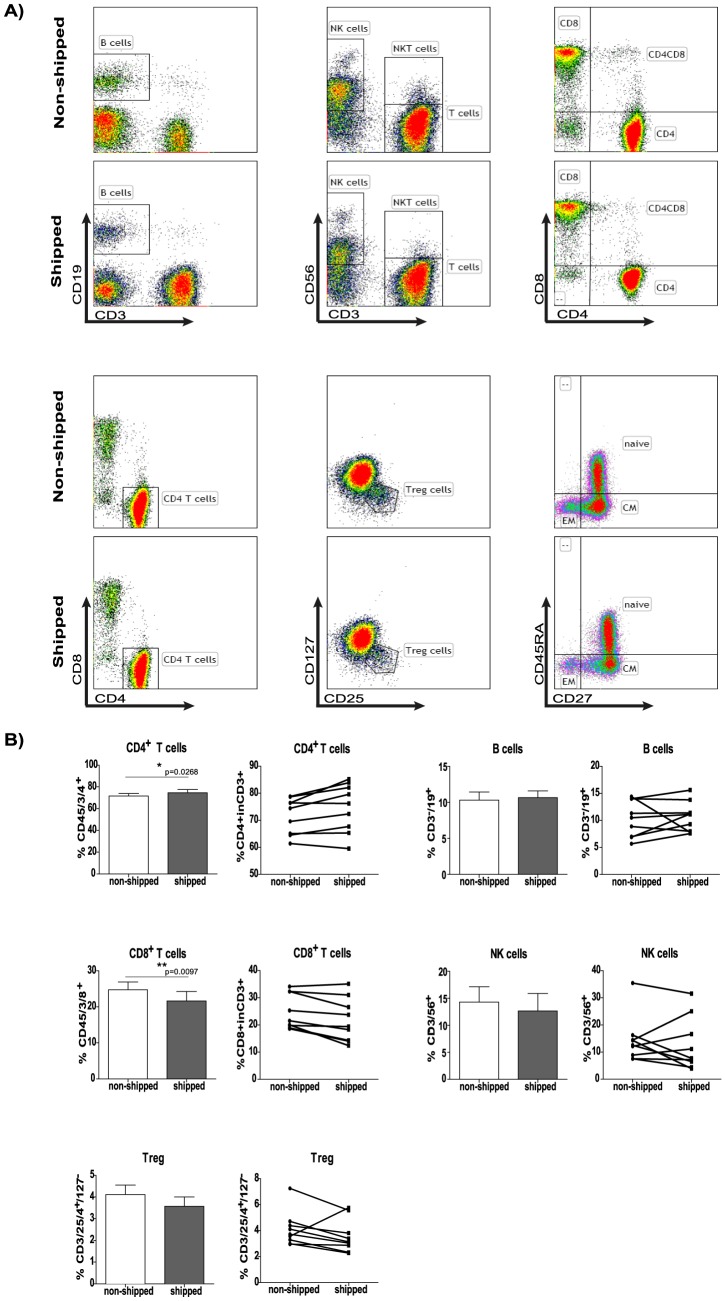
The effects of overnight shipping on lymphocyte subpopulations. PBMC were analyzed post cryo preservation by flow cytometry, to determine the *ex vivo* frequencies of CD3^+^CD4^+^ T cell, CD3^+^CD8^+^ T cell, CD3^+^CD127^−^CD25^+^CD4^+^ regulatory T cell, CD3^−^CD19^+^ B cell and CD3^−^ CD56^+^ NK cell populations. A) The gating strategy is presented on dot plots from one representative donor. Abbreviations: EM-effector memory cells, CM-central memory cells B) Bar graphs indicate the average frequencies for all tested donors (n = 9) in non-shipped vs. shipped samples. All graphs are shown with SEM values. Before-after graphs show the same data broken down for each individual.

We performed immune phenotyping combined with functional assays on cryopreserved PBMC (minimum 5 days post cryo preservation) and monitored the frequencies of major lymphocyte populations. We observed a transportation-induced alteration in CD4^+^/CD8^+^ T cell ratio with a mild but significant increase of CD4^+^ (3.9%) and decrease of CD8^+^ (12.7%) T cell percentages (p value: 0.0268 and 0.0097, respectively). In the case of B cells or NK cells, transportation-related changes were not significant however, notably in some individual cases an alteration of NK- cell frequencies could be observed (Fig. 2B, bar and dotted graphs).

Lymphocytes with regulatory properties such as Foxp3^+^CD25^+^CD4^+^ regulatory T cells are of crucial interest as they play essential role in maintaining peripheral tolerance preventing autoimmune diseases. The conventional identification of CD4^+^CD2^+^Foxp3 T_reg_ cells requires intracellular staining which includes permeabilization and fixation steps. It is known that fixation and permeabilization are rather “invasive” and consequently lead to several unspecific changes in the physical properties of the cells, thereby limiting the ability to make clear statements about shipment-induced changes. The cell surface expression of CD127 is known to correlate with the regulatory activity of CD4^+^CD25^+^Foxp3 T_reg_ cells as reduced expression of CD127 has been reported to define active T_reg_ cells with regulatory function [19], [20]. Therefore, to avoid intracellular staining we combined this marker with other known regulatory T cell markers (CD4, CD25), to most precisely define T_reg_ cells without the necessity of intracellular Foxp3 staining [21]. Using this strategy we observed a tendency towards decrease in T_reg_ frequencies on individual levels while, comparing pooled data of transported vs. non-transported groups (see Fig. 2B bar graphs) revealed only a mild decrease in T_reg_ frequencies, which was statistically not significant. (Fig. 2B).

### Effect of shipping on lymphocyte functions

**Figure 3:**
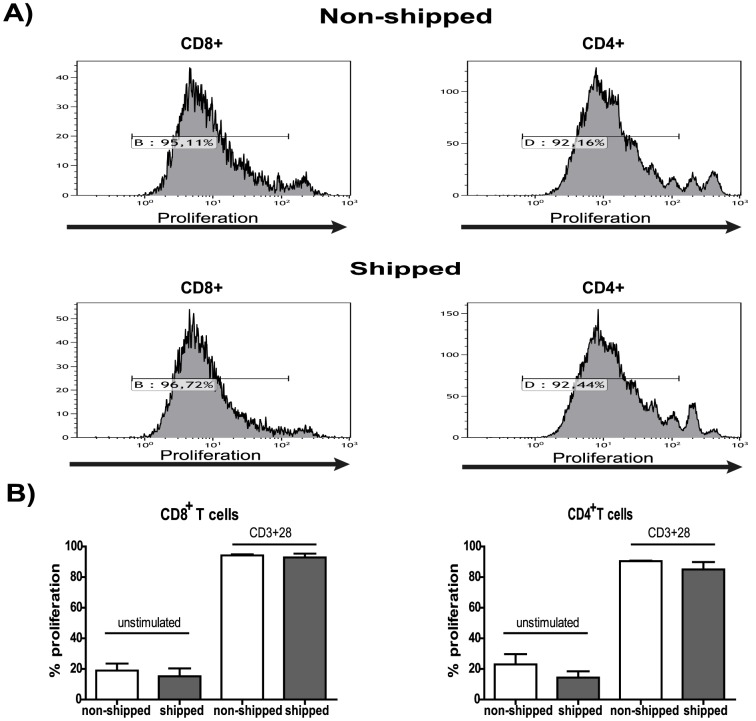
The effects of overnight shipping on T cell proliferation. PBMC from non-shipped vs. shipped samples were thawed, loaded with eFluor670 cell proliferation dye and stimulated with plate bound anti-CD3 (OKT3) and soluble anti-CD28 antibodies for 6 days (as described in the methods). Cells were harvested and proliferation was analyzed by flow cytometry. A) A representative graph is shown for CD8^+^ and CD4^+^ T cell proliferation. B) Bar graphs summarize the percentage of proliferating CD8^+^ (left) and CD4^+^ (right) T cells of all donors (n = 9).

Next we addressed the question of whether transportation affects functional capacity of particular immune cell subsets. To best cover the major domains of T cell function we looked at T cell proliferation, cytokine production and T cell receptor- and IL-6 mediated signaling. To do so, we first performed basic *in vitro* T cell proliferation assays using PBMC derived from non-shipped vs. shipped blood samples and observed that the proliferative capacity after anti-CD3 + anti-CD28 stimulation of both CD4^+^ and CD8^+^ T cells was fully preserved upon transportation (Fig. 3A and B).

**Figure 4:**
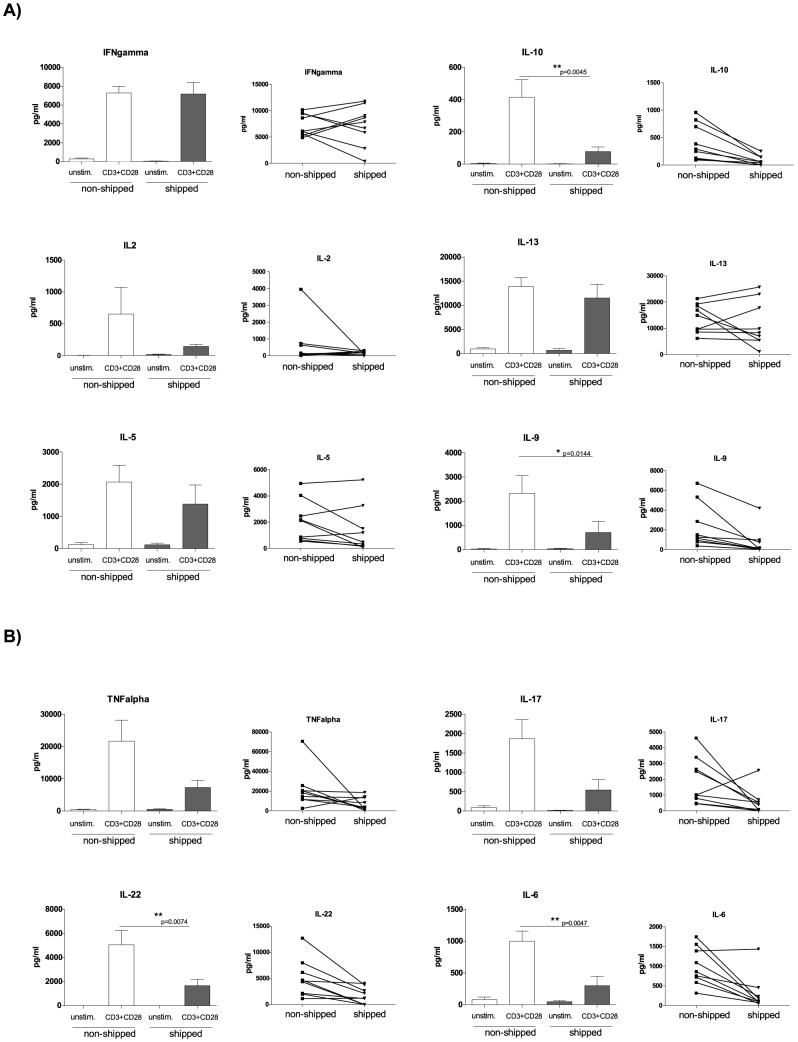
The effects of overnight shipping on cytokine production. PBMC were thawed and *in vitro* stimulated (anti-CD3 + anti-CD28) for six days. Supernatants were collected from the plate-wells and cytokine levels were determined with cytokine bead array for all donors (n = 9). Bar graphs show unstimulated and stimulated conditions both for non-shipped and shipped samples. All graphs are shown with SEM values. Before-after graphs visualize the cytokine levels on individual level. A) Th1/Th2 cytokines; B) Th17 cytokines.

Besides proliferation, cytokine production is also a crucial component of effector T cell function. Therefore we also screened cytokine production and its alteration by transportation of blood samples prior to processing. Similar to the T cell proliferation assay, we stimulated total PBMC with anti-CD3 + anti-CD28 antibodies as a specific T cell trigger and subsequently determined cytokine levels in the cellular supernatants. Although we could not observe a global shipment-induced alteration in ability of cytokine production we identified a group of “shipment sensitive” cytokines. These included IL-10, IL-22 (reduced in all tested donors) and IL-17, IL-9 and IL-6 (reduced in eight out of nine donors). Furthermore, we found another group of cytokines including IFNγ, IL-2, IL-5, TNFα and IL-13 the production of which seemed to be randomly influenced by shipment within the tested donor population (Fig. 4). To clarify if pre-processing delay might lead to these changes, we tested some representative cytokines from both groups (shipping sensitive: IL-10, IL-17, IL-6 and randomly influenced: INFγ and TNFα). The intra-individual values were relatively stable in non-shipped samples processed within 2 hours vs. 24 hours (S2 Supporting Information). No significant differences could be detected in the levels of the tested cytokines. Notably, INFγ levels were increased in case of delayed processing in all individuals (n = 4, p value: 0.0516; S2 Supporting Information), while shipping induces a rather individual-dependent alteration (Fig. 4A).

**Figure 5:**
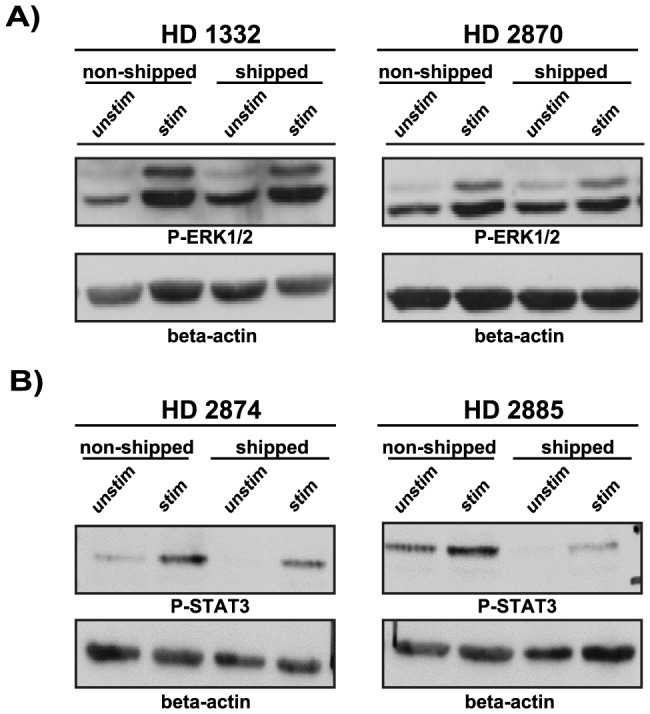
The effect of overnight shipment on signal transduction machinery. PBMC were stimulated with anti-CD3 (OKT3) + anti-CD28 antibodies for 5 min (A) or with rhIL-6 for 30 min (B) at 37°C in RPMI medium and lysed immediately after stimulation. Post-nuclear lysates were subjected to SDS-PAGE and proteins were transferred onto nitrocellulose membrane. Subsequently, membranes were probed with anti-pERK (A) or with anti-p-STAT3 (Y705) (B) antibody. Equal protein loading was controlled by probing the membranes against β-actin. Results are shown in a form of scanned blots for 2 representative donors out of 4 tested individuals (for both signaling pathways).

Finally, we investigated T-cell function on the subcellular levels by monitoring TCR (T-cell receptor) and cytokine receptor signaling machineries. To do so, we determined the phosphorylation (activation) status of the downstream MAPK ERK1/2 and found that T cells isolated from transported blood remained fully responsive and the quality of the TCR signaling was also comparable to non-transported samples demonstrated by the equal phosphorylation of ERK in both sample groups. (Fig. 5A)

Besides TCR signaling, cytokine receptor pathways are also essential in modulating cellular immune responses. The TGF-β and IL-6 pathways modulate Th17 vs. T_reg_ decision-making and thereby are essential in fine-tuning and balancing pro- vs. anti-inflammatory responses [22]. Therefore we investigated if transportation would alter IL-6 signaling pathways by stimulating T cells with rhIL-6 and monitoring subsequent activation of the IL-6R signaling pathway determined by the phosphorylation of STAT-3, a major second messenger molecule downstream of IL-6R. We found that similar to TCR-mediated signaling the IL-6 receptor signaling machinery in shipped samples remained functionally intact and fully responsive to IL-6 stimulation but with somewhat reduced amplitude of phosphorylation compared to non-shipped samples (in three out of four samples) (Fig. 5B).

## Discussion

Clinical studies as well as diagnostic services often require transportation of blood samples from remote centers to appropriately equipped central laboratories, having trained personnel for monitoring phenotypic and functional parameters of lymphocytes. Despite the extensive usage of shipping services (overnight courier) the knowledge about the possible effects of blood transportation on subsequently isolated PBMC and the function of immune cell populations remains incomplete. We therefore investigated the effects of overnight blood transportation on the yield and survival (viability) of PBMC, the frequencies of T-, T_reg_-, B- and NK cells and functions of T lymphocytes. In order to best evaluate the effects of transportation (while excluding donor dependent variations) we used a cohort of healthy volunteers who donated blood twice: on site (non-shipped) and on a remote location followed by overnight transportation of the blood samples (shipped) to our laboratory. We demonstrate that transportation of blood samples in EDTA containing sampling tubes does not alter the yield and viability of isolated PBMC but results in a slight decrease in the recovery of cryopreserved PBMC upon thawing of shipped samples. The difference is statistically not significant, however higher sample numbers might result in a more pronounced difference. A reason for this phenomenon (also observed under certain ambient temperature conditions by Olson et al. [9]) could be that shipping-induced physical stress might render lymphocytes more sensitive to cryopreservation and/or the freeze-thaw procedure, (e.g. due to membrane damage) leading to an enhanced cell death. However this hypothesis remains to be elucidated.

Detailed immune phenotyping showed that the CD4^+^/CD8^+^ T cell ratio is mildly but significantly influenced by transportation effects, while minor, non-significant alterations could be observed in B cell frequencies and a more pronounced effect on NK cells when analyzed on individual subject level. The biological relevance of the mildly but significantly shifted CD4^+^/CD8^+^ T cell ratio is unknown, a detailed analysis of T-cell subpopulations and their ratio could clarify if there is any physiological impact of these changes. Furthermore, if multiple studies with larger healthy donor pool confirm these changes, the minimal shipping induced effect on CD4^+^ and CD8^+^ T cells can be taken into consideration when planning/analyzing immune monitoring studies thereby preventing false interpretations. Whether the altered B and NK cell frequencies are due to actual cell loss and/or caused by a down regulation of CD19 and/or CD56 needs also to be further investigated. Taken together, we suggest precaution to be taken and carefully choosing intra- and inter- subject controls when investigating B cell and NK cell populations in transported samples.

Due to their relevance in autoimmune diseases we also studied the frequencies of regulatory T cells (T_reg_ defined as CD3^+^CD127^−^CD25^+^CD4^+^) and observed a trend towards decrease in T_reg_ subpopulation of T cells in all but one donor (eight out of nine). Based on this observation we conclude that percentage of T_reg_ cells in different patient cohorts and/or samples are comparable only if they were subjected to the same transportation/cryopreservation conditions.

These observations cannot be explained by the pre-processing delay caused by shipping. In non-shipped samples processed with the same delay as shipped ones, we could not observe any significant changes or the tendency to decreased T_reg_ percentages. Also the stability of NK- and B- cell ratio was remarkable. (S1 Supporting Information)

Importantly T cell function remained intact in all subjects reflected by the equal ability of CD4^+^ and CD8^+^ T lymphocytes from shipped vs. non-shipped PBMC to proliferate. This is in full agreement with the unaltered TCR signaling machinery in T cells derived from transported blood samples. This result not only indicates that shipping does not generally alter proliferative capacity of T lymphocytes but also encourages us to suggest proliferation assays as a read out for test-runs when monitoring the quality of procedures on different sites and to test if the chosen transportation system is appropriate.

Pantaleo et al. suggested determination of IL-2 and IFNγ secretion as a minimal measure of T-cell functions for clinical monitoring of virus-associated diseases [23]. To our knowledge there is no comprehensive publication about cytokine production by PBMC derived from shipped samples. Therefore we set out to test the effect of transportation on production of a number of cytokines (Th1, Th2 and Th17) and observed a significant decrease in several cases (IL-10, IL-22, IL-9, IL-6), a statistically non-significant alteration, but clearly decreased tendency for IL-17, while the levels of others varied seemingly randomly in donor-dependent manner (IFNγ, IL-2, IL-5, TNFα, IL-13). This result indicates that transportation might not necessarily equally influence blood samples and donor dependent sensitivity might further influence the outcome of shipping-induced alterations in cytokine production. Of note, we investigated the cytokine production of PBMC (upon T-cell specific stimulation) thereby it is likely that other cell types (i.e. non-T cells) also contributed to the cytokine production-related observations. Importantly it has been shown earlier that cryopreservation itself does not influence monocyte- or T-cell- derived cytokine production [4], [24] and we could not detect the presented alterations in cytokine levels in non-shipped samples which had undergone the same pre-centrifuging delay as shipped samples (S2 Supporting Information), suggesting on one hand that the observed changes were induced exclusively by transportation and on the other hand that there is likely a donor dependent reaction to shipping condition. The background of this observation remains unclear and the question is beyond the scope of this study.

IL-6 cytokine receptor signaling is crucial to modulate pro- vs. anti-inflammatory decision making as it plays a role in balancing T_reg_ vs. Th17 differentiation program [25]. On the contrary to TCR signaling, the IL-6R signaling machinery was qualitatively influenced by transportation reflected by reduction in the total amplitude of phosphorylation of STAT3 in all analyzed samples. However, it is important to highlight that shipping did not result in a globally dysfunctional IL-6 receptor signaling machinery, as it remained inducible. At this point we cannot exclude that the observed reduction in activation level of STAT3 would lead to alteration in certain cellular functions and if yes to which extent. Higher number of individuals/experiments should be analyzed to reveal the level and the consequence of alterations in IL-6 mediated signaling machinery. Based on our results we suggest to take extra caution when investigating cytokine production or cytokine induced signaling pathways, using transported samples. We strongly advise local processing of blood samples and transportation of cryopreserved PBMC to a central laboratory for cytokine analysis or if it is not feasible (due to lack of devices, laboratory or trained personnel with experience in cellular research) to guarantee standardized shipping conditions to ensure comparable sample quality.

In essence our data show that transportation influences the quality of cellular immune biobanking and draws attention to the factors, which influence interpretation (and comparability) of subsequent analysis, as well as the dependence of the analysis from sample quality. While basic cell viability is not altered, the ratio of CD4^+^/CD8^+^ T-cell is affected and a subject-dependent effect can be observed for NK and B cells in some cases. Basic cell proliferation seems to be a robust read-out despite transportation, while cytokine measures and IL-6-mediated signal transduction show shipment related alterations. This has implications for trial design, endpoint analysis and measures to guarantee comparability of samples. Furthermore, as it is known that certain malignancies (e.g. cancer, HIV) alter the viability and functionality of PBMC [10], [26], [27] while others such as allergy, autoimmune diseases, infections, lymphoid malignancies influence the composition of PBMC subsets regardless of transportation conditions, inclusion of healthy donors as controls for the transportation process and its effects on the samples from each biomaterial collection site is highly recommended.

## Supporting Information

S1 Supporting Information **The effect of pre-processing delay on immune cell phenotypes.** PBMC were isolated within 2 hours (as the non-shipped samples) and within 21–22 hours (like the shipped samples, where the mean of pre-processing delay was 21.8 hours). PBMC were analyzed post cryo preservation by flow cytometry, to determine the *ex vivo* frequencies of CD3^+^CD4^+^ T cell, CD3^+^CD8^+^ T cell, CD3^+^CD127^−^CD25^+^CD4^+^ regulatory T cell, CD3^−^CD19^+^ B cell and CD3^−^ CD56^+^ NK cell populations. Bar graphs indicate the average frequencies for all tested donors (n = 4) in samples processed within 2 hours and with pre-processing delay (within 24 hours). All graphs are shown with SEM values. Before-after graphs show the same data broken down for each individual.
(JPG)

S2 Supporting Information **The effect of pre-processing delay on cytokine production.** PBMC were isolated within 2 hours (as the non-shipped samples) and within 21–22 hours (like the shipped samples, where the mean of pre-processing delay was 21.8 hours). PBMC were thawed and *in vitro* stimulated (anti-CD3 + anti-CD28) for six days. Supernatants were collected from the plate-wells and cytokine levels were determined with cytokine bead array for all donors (n = 4). Bar graphs show unstimulated and stimulated conditions both for samples processed within 2 hours and with pre-processing delay (within 24 hours). All graphs are shown with SEM values. Before-after graphs visualize the cytokine levels on individual level.
(JPG)
