# Supplementary figures and images for: Effects of Blood Transportation on Human Peripheral Mononuclear Cell Yield, Phenotype and Function: Implications for Immune Cell Biobanking
Source: PLoS One. 2014 Dec 26;9(12):e115920. doi: 10.1371/journal.pone.0115920 (PMC4277394; doi:10.1371/journal.pone.0115920)

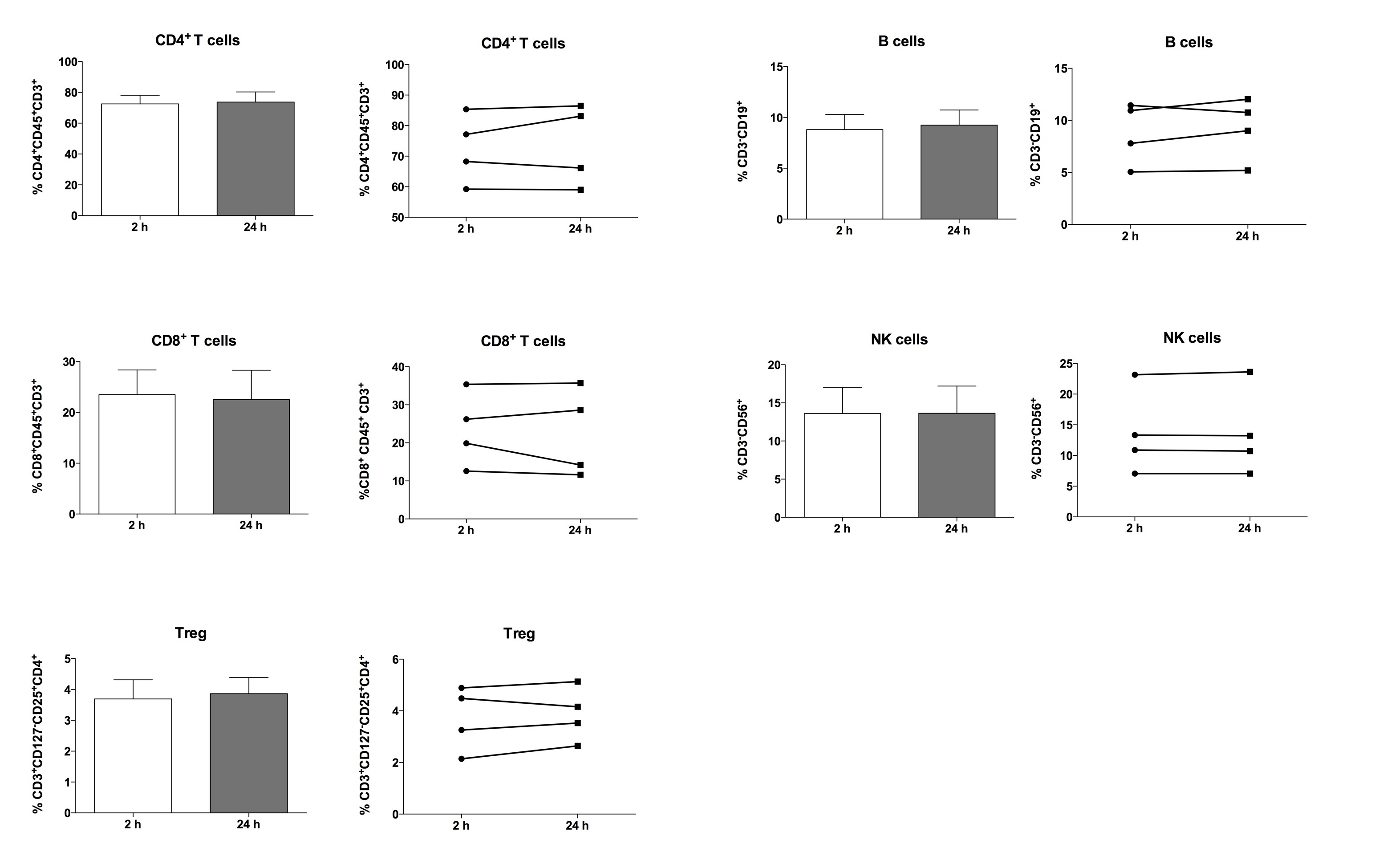

Supplement: S1 Supporting Information — The effect of pre-processing delay on immune cell phenotypes. PBMC were isolated within 2 hours (as the non-shipped samples) and within 21–22 hours (like the shipped samples, where the mean of pre-processing delay was 21.8 hours). PBMC were analyzed post cryo preservation by flow cytometry, to determine the ex vivo frequencies of CD3+CD4+ T cell, CD3+CD8+ T cell, CD3+CD127−CD25+CD4+ regulatory T cell, CD3−CD19+ B cell and CD3− CD56+ NK cell populations. Bar graphs indicate the average frequencies for all tested donors (n = 4) in samples processed within 2 hours and with pre-processing delay (within 24 hours). All graphs are shown with SEM values. Before-after graphs show the same data broken down for each individual. (JPG) [file pone.0115920.s001.jpg]

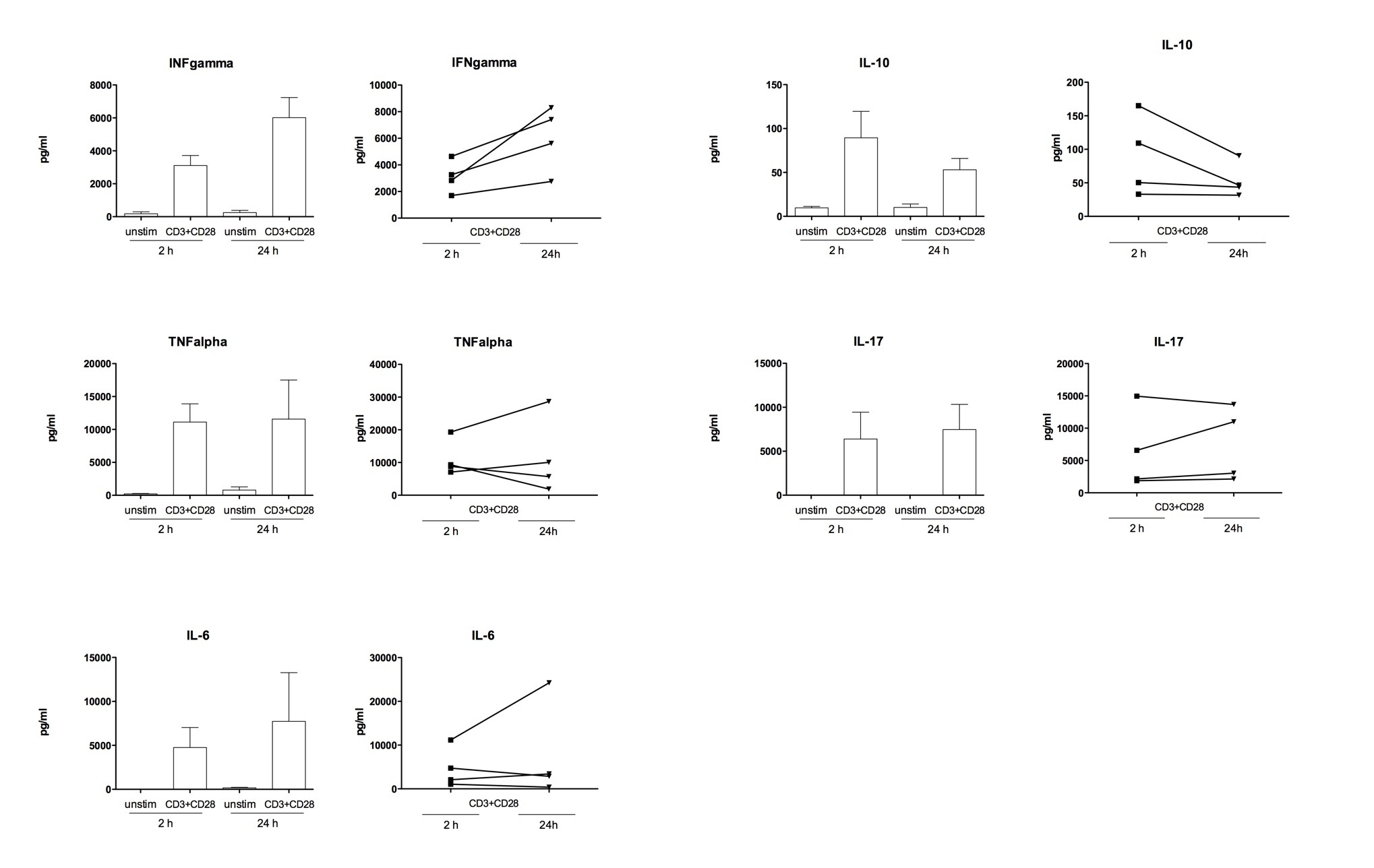

Supplement: S2 Supporting Information — The effect of pre-processing delay on cytokine production. PBMC were isolated within 2 hours (as the non-shipped samples) and within 21–22 hours (like the shipped samples, where the mean of pre-processing delay was 21.8 hours). PBMC were thawed and in vitro stimulated (anti-CD3 + anti-CD28) for six days. Supernatants were collected from the plate-wells and cytokine levels were determined with cytokine bead array for all donors (n = 4). Bar graphs show unstimulated and stimulated conditions both for samples processed within 2 hours and with pre-processing delay (within 24 hours). All graphs are shown with SEM values. Before-after graphs visualize the cytokine levels on individual level. (JPG) [file pone.0115920.s002.jpg]
